# Trends and distribution of birth asphyxia, Uganda, 2017-2020: a retrospective analysis of public health surveillance data

**DOI:** 10.4314/ahs.v25i2.33

**Published:** 2025-06

**Authors:** Allan Komakech, Freda L Aceng, Stella M Migamba, Petranilla Nakamya, Robert Mutumba, Lilian Bulage, Benon Kwesiga, Alex R Ario

**Affiliations:** 1 Uganda National Institute of Public Health, Kampala, Uganda; 2 Reproductive Health Department, Ministry of Health, Kampala, Uganda; 3 Clarke International University, Kampala, Uganda; 4 Department of Integrated Epidemiology, Surveillance and Public Health Emergencies, Ministry of Health, Kampala, Uganda

**Keywords:** Trends, Distribution, Birth asphyxia, Uganda

## Abstract

**Background:**

During 2018-2020, almost half of all neonatal deaths reviewed in Uganda were due to birth asphyxia. In 2015, Uganda adopted the Every Newborn Action Plan interventions to renew the focus on surveillance for birth asphyxia and other childhood-related illnesses. In 2016, the Ministry of Health implemented an evidence-based educational program for birth attendants about neonatal resuscitation techniques to improve the management of birth asphyxia. We described the trends and distribution of birth asphyxia in Uganda during 2017–2020 following these renewed efforts.

**Methods:**

We analysed birth asphyxia surveillance data from the District Health Information System 2 from January 2017–December 2020. We calculated the incidence of birth asphyxia per 1,000 deliveries at district, regional, and national levels. We used line graphs to demonstrate the trend of birth asphyxia incidence with the corresponding reporting rates at national and regional levels. We used logistic regression to evaluate the significance of the trends. Using choropleth maps, we described the distribution of birth asphyxia incidence at district level.

**Results:**

The average national annual incidence of birth asphyxia in 2020 was 31 per 1,000, with an increase of 4.5% from 2017 to 2020 (OR=1.05; 95%CI=1.04-1.05, p=0.001), with national quarterly reporting rates of 70-80% over the same period. Incidence in the Northern and Eastern Regions increased by 6% (OR=1.06; 95%CI=1.05-1.07, p=0.001) and 5% (OR=1.05; 95%CI=1.03-1.05, p=0.001), respectively, over the study period. Bundibugyo, Iganga, and Mubende Districts had rates of >60/1,000 during each of the four years of the study period. The least affected district was Kazo District, with an overall incidence of 3/1,000 over the study period.

**Conclusion:**

The incidence of birth asphyxia increased nationally from 2017-2020. We recommend efforts towards reducing the burden of birth asphyxia in Uganda, with emphasis on the most affected districts.

## Introduction

The World Health Organization (WHO) defines birth asphyxia as the failure to initiate and sustain breathing at birth^1^. Birth asphyxia is a leading cause of brain damage among newborn children, with up to 80% of survivors suffering from disabilities, developmental delays, cerebral palsy, intellectual disabilities, and behavioural problems^2^–^4^. Worldwide, birth asphyxia is responsible for an estimated 900,000 neonatal deaths per year^5^. Birth asphyxia incidence in developing countries has previously been reported to be 10 times that in developed countries^6^. Studies done in Bangladesh^7^ and Nigeria^8^ observed that birth asphyxia was responsible for 39% and 23.3%, respectively, of all neonatal deaths.

Risk factors for birth asphyxia are grouped according to whether they occur before birth (antepartum risk factors), during birth (intrapartum risk factors), or after birth (postpartum risk factors). Antepartum risk factors include severe maternal hypotension or hypertensive diseases during pregnancy, history of stillbirth, young maternal age, and advanced maternal age. Intrapartum risk factors include malpresentation of the fetus, a prolonged second stage of labour, and home delivery. Postpartum risk factors include low birth weight, high birth weight, preterm delivery, and poor resuscitation efforts^9^–^12^. The majority of these are preventable, as evidenced by the regional variations across the world^13^.

During 2018-2020, almost half of all neonatal deaths reviewed in Uganda were due to birth asphyxia^14^. Studies done in Uganda implicated antepartum and intrapartum risk factors, including complexities of referral systems, non-attendance of antenatal care by mothers, knowledge gaps among health workers, lack of equipment, and high health worker-to-patient ratio as the major culprits^15^–^17^. Furthermore, in 2020, the COVID-19 pandemic emerged^18^ around the world with Uganda also being affected. This might have led to an increase in cases in 2020 as witnessed in some studies^19^. In 2015, Uganda adopted the Every Newborn Action Plan (ENAP)^20^, which included a renewed focus on the surveillance of birth asphyxia cases and the development of a national strategic plan for managing birth asphyxia and other childhood illnesses. The ENAP is a global initiative developed by the World Health Organization (WHO) and the United Nations Children's Fund (UNICEF). Before this, the Millennium Development Goals were set but although they focused on child mortality, neonatal mortality remained a great challenge, thus justifying the ENAP. The scope of the ENAP was to improve health systems, promote enhanced care for small and sick newborns, and improve the quality of care for newborns, with specific targets for 2020, 2025, and 2030 set^20^. The ENAP targeted multiple levels of care including primary care and community level through educating families and communities about newborn care, promoting healthy behaviours, and providing support for home-based care practices; health facility level through strengthening the capacity of health facilities to deliver quality care during childbirth and for sick and small newborns and policy and system level through advocating for stronger policies, strategies, and financing for maternal and newborn health to ensure sustainable impacts^20^.

Furthermore, in 2016 the Ministry of Health rolled out the Helping Babies Breathe (HBB) initiative, meant to improve the prevention and management of birth asphyxia by health workers. However, the impact of these interventions on birth asphyxia incidence was unknown. We described the trends and distribution of birth asphyxia in Uganda during 2017-2020, the era following these renewed efforts, to understand the impact of these interventions and the COVID-19 pandemic on birth asphyxia incidence in Uganda.

## Materials and Methods

### Study setting

Uganda is located in East Africa with an estimated population of 41.6 million people^21^. There are four regions (Central, Western, Northern, and Eastern) and 15 sub regions (Acholi, Ankole, Bugisu, Bukedi, Bunyoro, Busoga, Kampala, Karamoga, Kigezi, Lango, North Central, South Central, Teso, Tooro, WestNile) in Uganda. The country has a total of 135 districts and 11 cities that are grouped into the four regions named above^21^. The health system comprises public and private health sectors and healthcare is provided through a decentralized system in which services are delivered across seven tiers. These include national referral hospitals, regional referral hospitals, district hospitals, Health Centres IV (HCIV), Health Centres III (HCIII), Health Centres II (HCII), and community health workers locally referred to as the Village Health Teams (VHTs)^22^. In Uganda, birth asphyxia is a condition that can be managed at HCII facilities and above. This is principally due to the care package such as trained and specialized health workers, and advanced medical equipment that are available at the HCIIs, HCIIIs and HCIVs.

### Study design and data source

We conducted a nationwide surveillance data analysis of birth asphyxia cases from 2017 to 2020 using data abstracted from the Uganda District Health Information System 2 (DHIS2), a web-based reporting tool introduced to Uganda in 2012. Data on birth asphyxia data are routinely generated at health facilities using the integrated maternity register, MCH 006.

The data from these registers are aggregated into a health facility monthly report (paper form) which is submitted to the health sub-district and then to the district health offices. At the district health office, data from the paper-based reports are entered into DHIS2. Data in DHIS2 are then grouped at national, regional, district, sub-county, and facility levels. These data are received from public, private for profit and private not for profit health facilities registered by the Ministry of Health. At the national level, the Reproductive and Infant Health Department of the Ministry of Health and other stakeholders use data from DHIS2 to make decisions and plan interventions on reproductive and infant health.

### Study variables, data abstraction and analysis

We abstracted data from DHIS2 using data elements in the HMIS105 outpatient register that contains data on both birth asphyxia and total deliveries. Data abstraction was conducted by two trained data analysts who meticulously extracted the relevant data from both versions of the DHIS2 system. This process ensured the consistency and accuracy of the data used for the analysis. The lead investigator supervised the analysts to maintain data integrity throughout the abstraction process.

Data elements/variables used were from the older and the newer version of DHIS2. In the older version, we used “105-2.2 Birth Asphyxia” and “105-2.2a Deliveries in unit” to identify birth asphyxia cases and total deliveries respectively. In the newer version of DHIS2, we used “105-MA24. No. of babies with Birth asphyxia” and “105-MA04a. Deliveries in unit-Total” to identify birth asphyxia cases and total deliveries respectively. The data from the two versions of DHIS2 were downloaded, merged and summarized in Microsoft Excel sheets. We calculated national quarterly reporting rates of birth asphyxia by dividing the available monthly reports per quarter for all districts by the expected monthly reports for all districts per quarter. We abstracted data for birth asphyxia cases and total deliveries during 2017–2020 from the DHIS2. We disaggregated the data into national, regional, and district levels. We calculated the annual incidence rates per 1,000 for birth asphyxia cases at national, regional, and district levels by dividing the total birth asphyxia cases during the year by the total deliveries during that year and multiplying by 1,000. We obtained the mean annual incidence rates for the national and regional levels by adding annual incidence rates for the four years of study and dividing by four.

We plotted the quarterly mean incidence of birth asphyxia against the study period in years to present the trend in incidence at national and regional levels from 2017–2020 and used logistic regression to establish the significance of the trends. We also used choropleth maps generated using Quantum Geographic Information System (QGIS) to present the district distribution of the birth asphyxia incidence across the country.

## Results

Trend of annual incidence rate of birth asphyxia, national level, Uganda, 2017–2020

In Uganda, there were a total of 4,625,336 deliveries and 134,801 birth asphyxia cases during 2017-2020. The average national incidence of birth asphyxia over the four years in Uganda was 29 per 1,000 deliveries. The highest annual incidence (32 birth asphyxia cases per 1,000 deliveries) over the four years was recorded in 2020.

**Figure 1:**
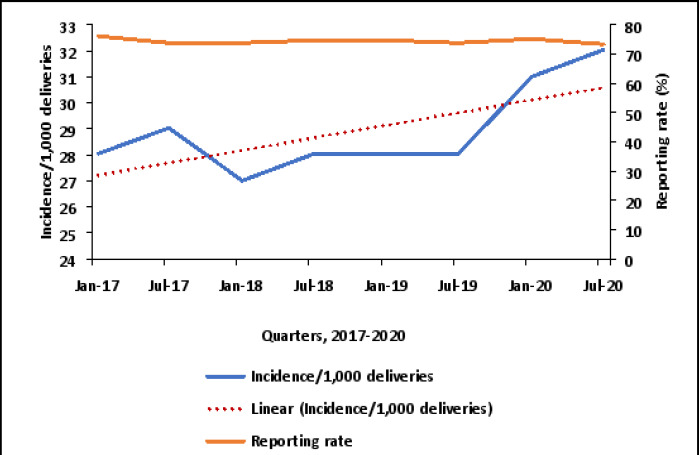
Quarterly trends of birth asphyxia incidence/1,000 total deliveries, Uganda, 2017–2020

Figure 1 shows an increasing trend of birth asphyxia from 2017–2020. The incidence of birth asphyxia increased from 2017–2020 and this was tested and found to be statistically significant (OR 1.045; 95% CI 1.04, 1.05). The highest quarterly incidence rates were observed in 2020 (Figure 1), with the highest incidence over the four years registered in 2020 at 32 per 1000. Reporting rates remained fairly stable (between 70% and 80%) from January 2017 to December 2020 (Figure 1).

### Trend of annual incidence rate of birth asphyxia cases, regional level, Uganda, 2017-2020

The increase in the incidence rates of birth asphyxia/1,000 total deliveries were noted in the Northern Region (OR 1.07; 95% CI: 1.05-1.08) indicating an increase of 7%; and in the Eastern Region (OR 1.05; 95% CI: 1.04-1.06) indicating an increase of 5% (Table 1). The incidence rates in other regions remained unchanged (Table 1).

**Table 1:** Significance of trends of birth asphyxia incidence at regional level, Uganda, 2017–2020

Region 2017/2020	Odds Ratio	95% CI	P-Value
**Northern**	1.07	1.05-1.08	<0.001*
**Eastern**	1.05	1.04-1.06	<0.001*
**Central**	1.00	0.99-1.01	0.54
**Western**	0.99	0.99-1.01	0.75

* indicates p value <0.05

Central Region registered the highest mean annual incidence rate of 30 birth asphyxia cases/1,000 total deliveries and Northern Region registered the lowest mean incidence rate of 28 per 1,000 deliveries over the four years.

### Spatial distribution of birth asphyxia incidence rates, district level, Uganda, 2017-2020

**Figure 2:**
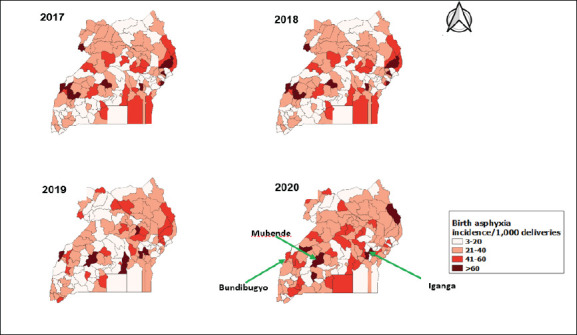
Spatial distribution of birth asphyxia cases/1,000 total deliveries, Uganda, 2017–2020

There was minimal spatial clustering of high-incidence districts for birth asphyxia during 2017-2020. However, specific districts had persistently high birth asphyxia incidence rates over the study period; these included Bundibugyo, Mubende, and Iganga (Figure 2).

## Discussion

Birth asphyxia incidence rates increased significantly in Uganda during 2017-2020, specifically due to increases in the Northern and Eastern Regions. Spatial trends showed minimal clustering of birth asphyxia incidence in the country. However, specific districts faced persistently high birth asphyxia incidence rates over the four years.

While the reasons for the increase in birth asphyxia incidence over the study period is not clearly understood, the burden of birth asphyxia is particularly high in East and Central Africa compared to other regions of Sub-Saharan Africa^23^,^24^. This is due to poor obstetrics coverage, inequity and inequality resulting from gaps in local health financing models, inaccessible health facilities, socio-cultural norms, low literacy levels, shortage in health workers and supplies, low level of health service utilization and inadequate resource allocation to healthcare^24^–^26^. Funding health services can lead to provision of services vital to reduce and prevent birth asphyxia.

The highest incidence of birth asphyxia during the study period occurred in 2020 at 32 birth asphyxia cases per 1,000 deliveries compared to 28/1000 total deliveries in the three previous years. The is likely due to the delayed access of pregnant women to health facilities following the imposition of the COVID-19 lockdown travel restrictions^27^. A study in a rural hospital in Central Uganda showed an increase of 7% in birth asphyxia incidence during the early phase of the COVID-19 lockdown versus before the lockdown^19^. The COVID-19 pandemic caused massive disruptions throughout many Africa countries affecting access to health facilities as evidenced in studies in Malawi, Ethiopia and Ghana^28^–^30^. Furthermore, crisis situations do not necessarily lead to reduction in reproduction^31^ and yet access to health services is greatly affected during such periods^31^. Special considerations should therefore be ensured to facilitate the access of pregnant women to health facilities during lockdown situations to improve access to health care.

While several districts had intermittently high rates of birth asphyxia, Bundibugyo, Iganga, and Mubende districts had persistently high incidences of >60 cases per 1,000 deliveries. This is approximately twice the national average rate. Kazo District was the least affected district with 3 cases of birth asphyxia/1,000 deliveries. Although no particular reason (socioeconomic, health services distribution or otherwise) can be identified to explain the non-clustered patterns of birth asphyxia in Uganda, all regions of Uganda have their fair share of poverty^32^, issues with access to- and availability of health facilities^33^, and differing cultural and social norms, known factors that can lead to birth asphyxia. Studies done to establish socio-demographic and health facility factors associated with birth asphyxia, particularly in the highly affected regions would be beneficial.

## Limitations

Secondary data in DHIS2 is limited in terms of variables to provide a sufficient assessment of birth asphyxia incidence in Uganda. Studies using primary data to determine associated factors may be more beneficial in understanding increasing birth asphyxia trends in Uganda. This will help to improve already-existing evidence-based interventions. Secondly, given the low average reporting rates over the study period (<80%), a true representation of birth asphyxia incidence might be limited. It should also be noted that since some deliveries occur outside the hospital, it is possible that the incidence rates we are reporting here are an underestimate. Lastly, we did not assess for representativeness of the data, being from public, private for profit or private not for profit; therefore, our data should be interpreted in this context and future studies done to have this component.

## Conclusion

Birth asphyxia incidence increased over the four years of our study period despite a decrease in reporting rates. The highest incidence over the four years was recorded in 2020. Bundibugyo, Iganga, and Mubende districts had a persistently high birth asphyxia incidence (>60/1,000 deliveries). Kazo district was the least affected district (3/1000 deliveries).

We recommend efforts towards reducing the burden of birth asphyxia in Uganda, with emphasis on the most affected districts by studying factors leading to birth asphyxia in the specific districts. Furthermore, we recommend continuous analysis of surveillance data on birth asphyxia to understand the impact of the COVID-19 pandemic on birth asphyxia incidence in Uganda.
